# Ease and difficulty of pre-hospital airway management in 425 paediatric patients treated by a helicopter emergency medical service: a retrospective analysis

**DOI:** 10.1186/s13049-016-0212-9

**Published:** 2016-03-05

**Authors:** Alexander R. Schmidt, Lea Ulrich, Burkhardt Seifert, Roland Albrecht, Donat R. Spahn, Philipp Stein

**Affiliations:** Department of Anaesthesiology, University Children’s Hospital, Zurich, Switzerland; Institute of Anaesthesiology, University and University Hospital Zurich, Zurich, Switzerland; Department of Biostatistics, Epidemiology, Biostatistics and Prevention Institute, University of Zurich, Zurich, Switzerland; Swiss Air-Ambulance, Rega (Rettungsflugwacht/Guarde Aérienne), Zurich, Switzerland

**Keywords:** Paediatric airway, Pre-hospital airway, Emergency airway, Endotracheal tube size and depth, HEMS

## Abstract

**Background:**

Pre-hospital paediatric airway management is complex. A variety of pitfalls need prompt response to establish and maintain adequate ventilation and oxygenation. Anatomical disparity render laryngoscopy different compared to the adult. The correct choice of endotracheal tube size and depth of insertion is not trivial and often challenged due to the initially unknown age of child.

**Methods:**

Data from 425 paediatric patients (<17 years of age) with any airway manipulation treated by a Swiss Air-Ambulance crew between June 2010 and December 2013 were retrospectively analysed. Endpoints were: 1) Endotracheal intubation success rate and incidence of difficult airway management in primary missions. 2) Correlation of endotracheal tube size and depth of insertion with patient’s age in all (primary and secondary) missions.

**Results:**

In primary missions, the first laryngoscopy-guided endotracheal intubation attempt was successful in 95.3% of cases, with an overall success rate of 98.6%. Difficult airway management was reported in 10 (4.7%) patients. Endotracheal tube size was frequently chosen inadequately large (overall 50 of 343 patients: 14.6%), especially and statistically significant in the age group below 1 year (19 of 33 patients; *p* < 0.001). Tubes were frequently and distinctively more deeply inserted (38.9%) than recommended by current formulae.

**Conclusion:**

Difficult airway management, including cannot intubate and cannot ventilate situations during pre-hospital paediatric emergency treatment was rare. In contrast, the success rate of endotracheal intubation at the first attempt was very high. High numbers of inadequate endotracheal tube size and deep placement according to patient age require further analysis. Practical algorithms need to be found to prevent potentially harmful treatment.

## Background

Pre-hospital paediatric airway management is complex. Different pitfalls such as anatomical airway obstruction (poorly positioned head, inappropriate facemask usage or tonsillar hypertrophy) and functional airway obstruction (laryngospasm, bronchospasm or opioid induced thorax rigidity) need to be quickly recognized during bag mask ventilation. Prompt response to these problems is necessary to maintain adequate ventilation and oxygenation due to the known low functional residual capacity in new born and young children resulting in rapid hypoxaemia during apnoea [1, 2]. Regarding endotracheal intubation (ETI), a smaller oral cavity with a relatively large tongue, a more anterior larynx, a higher glottis and a longer epiglottis render laryngoscopy different compared to the adult anatomy [3].

Pre-hospital ETI by paramedics had high levels of misplacement (into the oesophagus or the hypopharynx) combined with a high mortality and morbidity rate [4], especially in the absence of end tidal carbon dioxide measurement [4–7]. Rates of successful ETI varied depending on the investigated patient group and the qualification of the intubating health care provider [8–12]. The ETI success rate for pre-hospital paediatric patients lies between 55 and 100% [13], with a high complication rate (unrecognised oesophageal intubation 14.6%, incorrect endotracheal (ET) tube size/depth of insertion 11–22%, cardiovascular collapse with consecutive need for resuscitation after ETI, potentially “lethal” ventilator settings 4.9%, inability to intubate 35%) in less experienced emergency medical service health care providers [14–16]. ETI can be more difficult in a pre-hospital setting, with a higher grading, according to Cormack and Lehane [17] and a higher incidence of difficult and failed laryngoscopy and airway management [18]. Others report pre-hospital ETI success rates are comparable to the in-hospital rate, especially if performed by highly skilled physicians [19].

The choice of the adequate ET tube size and depth of insertion is not trivial. Circumstances such as initially unknown age often jeopardize adequate airway management. In a former study, intubation depth in a helicopter emergency service (HEMS) was incorrect in 57% of paediatric ETI [20]. Since then, novel philosophies towards the use of cuffed paediatric ET tubes changed the practice among HEMS, making a new evaluation of the current routine necessary. Commonly used age-based formulae for ET tube size calculation are inappropriate in 20–30% of cases [21, 22]. With cuffed ET tubes it is possible to choose the correct ET tube size in almost 100% of patients, if the child’s age is known [23–25]. Small for age ET tube sizes can lead to an increase in airway resistance, but may have to be chosen intentionally if a narrow airway is clinically expected (oedema, trauma). If chosen too large, ET tubes may lead to pressure points on the tracheal mucosa followed by oedema or necrosis [22, 26, 27]. Correct intubation depth is critical as small amounts of motion can lead to supraglottic or endobronchial misplacement [28–30]. Head-neck flexion moves the ET tube further down into the trachea (9.7 to 20 mm), whereas 30° head-neck extension pulls the ET tube back (9.8 to 22 mm) [28]. The consequence is a small margin of safety concerning depth of ET tube placement. Clinical assessment of the correct ET tube depth is difficult without the help of concluding imaging, because auscultation can be misleading and inconclusive in a noisy pre-hospital setting. Defining correct intubation depth solely on formula based calculations can lead to incorrect placement [31, 32]. A study by Weiss et al. pinpointed that only visual placement of the Microcuff ET tube marking between the vocal cords leads to a correct tracheal positioning of the ET tube tip in all of the investigated children in an in-hospital setting [31, 33].

The aims of this study were to evaluate the incidence of difficult airways and the ETI success rate on primary missions and to monitor ET tube size and depth of insertion in paediatric patients treated by a Swiss HEMS.

## Methods

### Ethics

The data analysis was approved by the local ethics committee (Kantonale Ethikkommission Zurich, Switzerland, KEK-ZH-2014-0067).

### Study design and participants

Retrospective observational study including all paediatric patients (< 17 years of age) with any airway manipulation (bag mask ventilation, ETI, supraglottic airway or tracheotomy) treated by a Swiss Air-Ambulance (Rega) crew between June 2010 and December 2013.

### Setting

The Swiss Air-Ambulance (Rega) is a non-profit HEMS that performs more than 11.000 emergency missions per year from 12 bases and one partner-base in Switzerland. Operation profiles comprise primary missions (scene to hospital) and secondary missions (hospital to hospital) with all types of emergencies (medical, trauma and evacuations) and patient characteristics. Patients transported from hospital to hospital (secondary mission) in this study, had already been intubated by the referring hospital staff prior to our HEMS transfer.

The Rega HEMS crew consists of a helicopter pilot, a paramedic and a specially trained emergency physician. These physicians are to be on the anaesthesia track (> 1 year), experienced in emergency medicine (> 4 years in total, advanced cardiovascular and trauma life support providers approved by the American Heart Association or the European Resuscitation Council), paediatric anaesthesia (paediatric advanced life support providers), and intensive care (3–6 months). The Rega standard advanced paediatric airway management is ETI performed as a controlled rapid sequence induction and intubation without obligation to omit mask ventilation prior to ETI as described by Neuhaus et al. [34]. The equipment is standardised throughout the entire organisation. Available neuromuscular blocking agents are rocuronium bromide and suxamethonium chloride. Rega used Microcuff ET tubes (Kimberly-Clark Health Care Europe, Zaventem, Belgium) with internal diameters from 3 to 5 mm, in 0.5 mm increments and Rusch super safety clear tubes (Teleflex Medical Europe Ltd, Athlone, Ireland) with internal diameters of 6–8 mm respectively. All ET tubes in this study contained a cuff. Laryngeal masks unique™ (LMA, Bonn, Germany) were used by the Rega as primary supraglottic (alternative) airway devices. In this study, also laryngeal tubes (VBM Germany, Sulz a.N., Germany) were used by first responders prior to Rega arrival.

### Descriptive variables

Demographic data of the patient (age and gender), mission characteristics (primary and secondary), emergency specifications (national advisory committee for aeronautics (NACA) score, Glasgow coma scale (GCS) and type of emergency) and detailed information about airway management (indication, type of airway management, number of laryngoscopic attempts to accomplish ETI, ET tube size, depth of insertion, use of alternative airway equipment, use of capnography and neuromuscular blocking agents) were recorded.

### Endpoints and outcome variables

Endpoints were: 1) ETI success rate and incidence of difficult airway management reported by the treating physician in primary missions. 2) Correlation of ET tube size and depth of insertion with patient’s age in all (primary and secondary) missions. ETI was successful if expiratory carbon dioxide was detected by capnography.

Throughout this manuscript we used a 15% tolerance concerning ET tube size and depth of insertion as published data shows this relative distance from the ET tube tip to the carina in the shortest trachea per age group [31]. The adequate ET tube size according to patient’s age was defined by the manufacturer (Kimberly-Clark Health Care Europe, Zaventem, Belgium). Thus (including the tolerance) until the age of 8 months, only a 3.0 ET tube size was judged as adequate, from 8 months until 14 years of age the recommended size +/− 0.5 mm, and from 14 to < 17 years the recommended size +/− 1 mm was judged as adequate. ET tube depth of insertion was measured from the front teeth to the tube tip. The measured value was compared to age-based calculations by Weiss et al. (10.612 + age [years] × 0.5493 in cm) [31] and a standard formula for oral ET tube depth of insertion (12 + age [years] × 0.5 in cm) for children over 2 years of age [35].

### Data collection

Relevant missions were identified in the Rega database. Data on mission and patient characteristics was extracted from that database. Thereafter, the corresponding original HEMS protocols were analysed and data on airway management was extracted. The data was transferred into a spreadsheet (Microsoft Excel: mac 2011, version 13.5.3, Microsoft corporation, Redmond, USA).

### Bias

As obliged by the Swiss aviation authorities, the Rega database contains every helicopter movement. These movements are distinctively linked to mandatory information on patient’s characteristics and on the information about airway management, which limits the influence of selection bias. The analysed HEMS protocols were completed directly at the end of every mission by the responsible emergency physician, narrowing the effect of a recall bias.

### Statistical methods

Statistical data was analysed with the SPSS software (Version 22, IBM, Armonk NY, USA) in collaboration with the Division of Biostatistics from the Institute of Social and Preventive Medicine (University of Zurich). For non-normally distributed independent variables we used the “Wilcoxon-Mann–Whitney” test. A two-sided p-value of less than 0.01 was considered statistically significant. Chi square test was used to test independence of normally distributed data. Ordinal or skewed data was presented as median and interquartile range (IQR).

## Results and Discussion

In total, 4505 paediatric patients were treated by Rega HEMS crews during the study period. The study population consisted of 425 children with any airway manipulation. Of the performed helicopter missions, 225 (52.9%) were at the scene (primary) and 200 (47.1%) were secondary transports between hospitals of already ventilated patients (intubated prior to the mission). In all patients the method of airway management could be determined. Most commonly the children’s airway was secured with an ET tube. Capnography was used in all intubated patients to confirm ET tube position in the trachea. Details on patient, airway and mission characteristics are summarized in Table 1.

**Table 1:** Patient, mission and airway characteristics

	Primary mission	Secondary mission
Total (*n* = 425)	225	200
Gender		
Male	142 (63.1%)	115 (57.7%)
Age		
median (IQR)	6.4 (2.4–12.7)	5 (1.7–10.5)
NACA		
III	1 (0.4%)	1 (0.5%)
IV	31 (13.8%)	75 (37.5%)
V	116 (51.6%)	116 (58%)
VI	43 (19.1%)	8 (4%)
VII	34 (15.1%)	0
GCS		
< 9	206 (91.5%)	184 (92%)
> = 9	19 (8.5%)	16 (8%)
Trauma	151 (67.1%)	69 (34.5%)
Craniofacial injury	117 (77.5%)	55 (79.7%)
Other	34 (22.5%)	14 (20.3%)
Non-trauma	74 (32.9%)	131 (65.5%)
Neurological	20 (27%)	40 (30.5%)
Respiratory	37 (50%)	12 (9.2%)
Circulatory	13 (17.6%)	53 (40.5%)
Other	4 (5.4%)	26 (19.8%)
Airway (final)		
NIV	4 (1.8%)	5 (2.5%)
Tracheotomy (pre-existing)	3 (1.3%)	5 (2.5%)
Orotracheal int.	212 (94.2%)	172 (86%)
Nasotracheal int.	0	18 (9%)
Laryngeal mask	2 (0.9%)	0
Laryngeal tube	3 (1.3%)	0
CICV	1 (0.4%)	n/a
Neuromuscular blocking agents used (NACA III-V)	129/148 (87.2%)	n/a
Rocuronium	34 (26.4%)	
Suxamethonium	95 (73.6%)	
Neuromuscular blocking agents used (NACA VI-VII)	11/77 (13.3%)	n/a
Rocuronium	6 (54.5%)	
Suxamethonium	5 (45.5%)	

Summary of primary and secondary mission characteristics. Percentages are in reference to the main group (primary/secondary) or the subgroup respectively. *IQR* interquartile range. *NACA* national advisory committee for aeronautics. *GCS* glasgow coma scale. *NIV* non-invasive ventilation. *CICV* cannot intubate, cannot ventilate

### ETI success rate and incidence of difficult airway management in primary missions

In 215 patients an orotracheal on-scene ETI was attempted. The first laryngoscopy-guided ETI effort was successful in 205 (95.3%) patients. Difficult airway management was described by the treating physician in ten (4.7%) patients: Seven (3.3%) patients required 2–4 laryngoscopies until their trachea could be intubated (three resuscitations, four severe craniofacial injuries), resulting in a 98.6% overall ETI success rate. Cannot intubate (CI) situations were rare but present in three cases (1.4%), two of them because of blood in the traumatised airway. Both could be managed with a laryngeal mask. One cannot intubate, cannot ventilate (CICV) situation in a patient with obvious dysmorphia syndrome (Goldenhar) was encountered during an already long-lasting hypoxic cardiovascular resuscitation. Prolongation of the resuscitation efforts was judged as inadequate and the patient was declared dead.

### Correlation of ET tube size with patient’s age in all missions

**Fig. 1:**
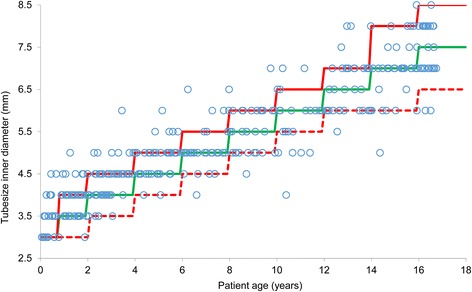
Chosen endotracheal tube (size of inner diameter in mm) for patient age (years). Adequate tube size for age is shown as the continuous green line, 15% upper tolerance limit as the continuous red line and 15% lower tolerance limit as the dashed red line. Adequate tube size in 82.5%, inadequately small in 2.9% inadequately large in 14.6%

Among all orotracheal intubated children (*n* = 384), ET tube size was noted in 343 (82.7%) of the protocols. Thereof, the chosen ET tube size was adequate in 82.5%, inadequately small in 2.9% and inadequately large in 14.6%, especially and statistically significant in the age group of new born to 1 year (19 of 33 children; *p* < 0.001; Fig. 1, Table 2). Children with an inadequate ET tube size were significantly younger than children with an adequate ET tube size: 3.4 (0.6 – 9.9) vs. 5.4 (2.8 – 13.5) years, (*p* < 0.001).

**Table 2:** Adequate oral endotracheal tube sizes according to sub groups

	Adequate	Inadequate (small)	Inadequate (large)	*p*-value
Gender				
Female (*n* = 133)	107 (80.5%)	6 (4.5%)	20 (15%)	*p* = 0.467
Male (*n* = 210)	176 (83.8%)	4 (1.9%)	30 (14.3%)	
Age in years				
0–1 (*n* = 33)	14 (42.4%)	0 (0%)	19 (57.6%)	*p* < 0.001
1–2 (*n* = 37)	30 (81.1%)	0 (0%)	7 (18.9%)	
2–4 (*n* = 56)	49 (87.5%)	0 (0%)	7 (12.5%)	
4–6 (*n* = 37)	32 (86.5%)	0 (0%)	5 (13.5%)	
6–8 (*n* = 32)	27 (84.4%)	1 (3.1%)	4 (12.5%)	
8–10 (*n* = 30)	27 (90%)	2 (6.7%)	1 (3.3%)	
10–12 (*n* = 21)	14 (66.7%)	5 (23.8%)	2 (9.5%)	
12–17 (*n* = 97)	90 (92.8%)	2 (2.1%)	5 (5.1%)	
Mission type				
Primary (*n* = 195)	165 (84.6%)	7 (3.6%)	23 (11.8%)	*p* = 0.253
Secondary (*n* = 148)	118 (79.7%)	3 (2.1%)	27 (18.2%)	
Medical indication				
Trauma (*n* = 194)	166 (85.6%)	6 (3.1%)	22 (11.3%)	*p* = 0.114
Non-trauma (*n* = 149)	117 (78.5%)	4 (2.7%)	28 (18.8%)	
Resuscitation				
Yes (*n* = 72)	53 (73.6%)	5 (6.9%)	14 (19.4%)	*p* = 0.035
No (*n* = 271)	230 (84.9%)	5 (1.8%)	36 (13.3%)	

Summary of adequate ET tube size (per age) according to subgroup. Endotracheal tube size copied from the original patient protocol. Adequate ET tube size is indicated by the Microcuff tube manufacturer. Outside a 15% tolerance, ET tube size is judged as inadequate. All ET tubes contained a cuff

### Correlation of ET tube depth of insertion with patient’s age in all missions

**Fig. 2:**
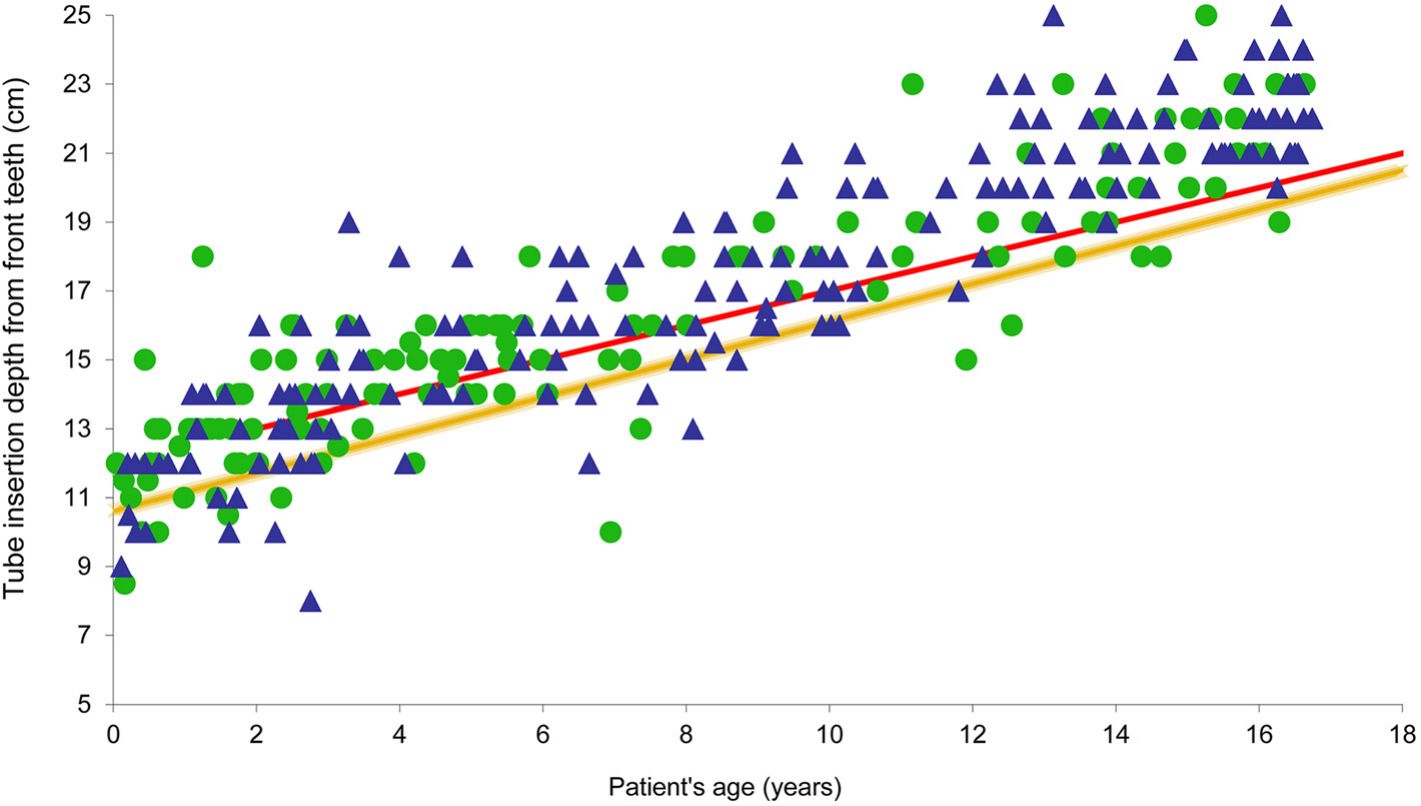
Tube depth of insertion (cm) for patient age (years). Filled triangle = primary mission; filled dot = secondary mission; yellow line = depth of insertion (10.612 + age [years] × 0.5493 in cm) according to Weiss et al. [31] Red line = depth of insertion according to formula (12 + [years] × 0.5 in cm) [35]

Orotracheal tube depth of insertion was noted in 303 (73%) of all protocols. Figure 2 shows depth of insertion of ET tubes for primary and secondary mission in relation to a calculated depth of insertion according to Weiss et al. [31] and to a standard formula for oral ET tube insertion for children over 2 years of age [35]. With regard to the depth of ETI calculated by the Weiss formula including a 15% tolerance, only six of 303 (2%) ET tubes were shallower, whereas 118 of 303 (38.9%) ET tubes were deeper than calculated. None of the ET tubes in this study had been placed too deep intentionally for reasons of lung separation or one lung ventilation.

## Discussion

This study investigated the incidence of difficult airways and the ETI success rate in primary missions and the choice of ET tube size and depth of insertion in all paediatric patients treated by a Swiss HEMS. The main findings were that during primary missions ETI was successful with the first attempt in 95.3%. Apart from that, ET tube sizes were chosen inadequately large in 14.6% of all cases, especially in the age group new born to 1 year, in which 57.6% of the ET tubes were chosen inadequately large for age. Depth of ET tube insertion was frequently and considerably deeper than calculated by formulae.

### Endotracheal intubation success rate and incidence of difficult airway management

Compared to Hansen et al. [13], our analysis showed a higher rate of successful ETI (> 95% at first attempt versus 81% in total). Our success rate is comparable to in-hospital paediatric emergency ETI [34]. Published studies concerning difficult airway management in adult patients, presented data similar to the findings in the current study regarding the incidence of a difficult airway and ETI. In a clinical setting, unexpected poor glottis visualisation during direct laryngoscopy was encountered in 1–9 % of intubation attempts, difficulties during laryngoscopy occurred in 5.8% and difficult ETI in up to 3.2% of all patients [10, 36, 37]. All patients in our study had a documented carbon dioxide measurement with any advanced airway device (supraglottic and ET tube) in place compared to less than 50% documented carbon dioxide measurement in the Hansen analysis [13]. We hypothesize that the difference in proficiency level (paramedic versus specially trained physician) and the mandatory use of capnometry to distinctively confirm ETI account for the discrepancy in terms of successful ETI.

In our HEMS organisation, supraglottic airway devices were only used as rescue devices in the case of inability to perform ETI. Questions concerning patient’s outcome after pre-hospital ETI versus supraglottic airways are still unanswered [38, 39].

### Correlation of endotracheal tube size with patient’s age

Choosing the adequate ET tube size can be difficult in emergency situations as formulae and the manufacturer recommendations are age dependant. Taking into account that this tube size recommendation for Microcuff tubes is correct in 97.4% of the cases (or infrequently needs an exchange with a smaller ET tube) [23], an incidence of 14.6% larger ET tubes than recommended even after adding a 15% tolerance predisposes a large group of patients to a risk of airway trauma.

During or after cardiopulmonary resuscitation, ET tube size was chosen inadequately in a higher (scantly not significant) proportion of cases. This may be due to a higher stress level or a shortage of time for the choice of correctly sized equipment. Interestingly, there was no difference between primary and secondary missions concerning adequate ET tube sizes. This may be a sign of a generally limited experience in advanced paediatric emergency airway management, especially in very young children.

### Correlation of endotracheal depth of insertion with patient’s age

Regarding the noted ET tube depth of insertion, most tracheas where intubated deeper than predicted by known age-based formulae, even after the addition of a 15% tolerance [31, 35, 40]. Keeping in mind that patients might be moved substantially during transport, incorrect depth of insertion predisposes to accidental bronchial intubation due to head inclination [28–30]. Only the placement of the depth marking of the correct Microcuff ET tube (Kimberly-Clark Health Care Europe, Zaventem, Belgium) for age between the vocal cords was accurate for all paediatric patients in contrast to age-based calculations [31, 33]. Hence a (supplemental) laryngoscopic inspection may be necessary to reposition the ET tube prior to transport.

Limitations of our study are its retrospective design, relatively low patient numbers in comparison to Hansen et al. [13] and the lack of visualisation of the ET tube depth of insertion by x-ray, fibre-optic inspection or ultrasound. Our original HEMS data was not standardised in terms of recording adverse events related to airway management, so the incidence would have been largely underestimated with limited scientific value and therefore this data was not presented and discussed in this study.

Nevertheless this is the largest analysis of pre-hospital paediatric emergency airway managements performed by designated pre-hospital emergency physicians only. Future prospective trials need be planned to assess adverse events and outcome after ETI of paediatric emergency patients by the hand of the experienced physician.

## Conclusion

Difficult airway management, including cannot intubate and cannot ventilate situations during pre-hospital paediatric emergency treatment was rare. In contrast, the success rate of ETI at the first attempt was very high. High numbers of inadequate ET tube size and deep placement according to patient age require further analysis. Practical algorithms need to be found to prevent inaccurate treatment. Capnography must be used during advanced airway management.
